# Supplementary material for: Does Food Intake of Australian Toddlers 12–24 Months Align with Recommendations: Findings from the Australian Feeding Infants and Toddlers Study (OzFITS) 2021
Source: Nutrients. 2022 Jul 14;14(14):2890. doi: 10.3390/nu14142890 (PMC9322480; doi:10.3390/nu14142890)
Supplement: Supplementary file 1 [file nutrients-14-02890-s001.zip › nutrients-1803227-supplementary.pdf]

Table S1. Daily serves of five food group and discretionary foods consumed by toddlers 12–<18 months stratified by breastmilk intake, OzFITS 2021.

Food Group	Consumers *n* (%)	AGHE Serve size [1]	Recommended serves per day	Number of serves per day [2]
		**Breastfed toddlers (*n* = 119)**		
Fruit	116 (97)	150 g	½	0.88 (0.45–1.47)
Vegetables	118 (99)	75 g	2-3	1.17 (0.54–2.23)
Cereals and grains	115 (97)	40 g bread	4	1.75 (0.87–2.56)
Meats and alternatives	111 (93)	65 g red meat	1	0.52 (0.22–0.95)
Dairy				
Without toddler milks	107 (90)	250 ml milk	1–1 ½	0.58 (0.21–1.21)
With toddler milks [3]	107 (90)	250 ml milk	1–1 ½	0.60 (0.21–1.21)
Fats and oils	69 (58)	7–10 g	1	0.15 (0–0.78)
Discretionary foods	101 (85)	418 kJ	0	0.73 (0.22–1.76)
		**Non-breastfed toddlers (*n* = 114)**		
Fruit	108 (95)	150 g	½	1.15 (0.69–1.63)
Vegetables	111 (97)	75 g	2–3	1.55 (0.60–2.28)
Cereals and grains	110 (96)	40 g bread	4	2.21 (1.33–3.20)
Meats and alternatives	102 (89)	65 g red meat	1	0.47 (0.18–1.01)
Dairy				
Without toddler milks	113 (99)	250 mL milk	1–1 ½	1.56 (0.79–2.24)
With toddler milks [3]	114 (100)	250 mL milk	1–1 ½	2.24 (1.55–2.96)
Fats and oils	67 (59)	7–10 g	1	0.10 (0–0.51)
Discretionary foods	100 (88)	418 kJ	0	0.79 (0.20–2.04)

[1] AGHE; Australian Guide to Healthy Eating; Equivalent of 40 g bread, 65 g red meat, or 250 ml milk. For example, 40g of cheese equals one serve of dairy [1]. [2] Values are median (IQR). The lower bound is used where a range exists for a recommended serves per day. [3] Dairy, including formula/toddler milk. A serve of formula/toddler milk is defined as 250 mL prepared volume. OzFITS, Australian Feeding Infants and Toddler Study.

Table S2. Energy and percentage total energy from five food group and discretionary foods for toddlers aged 12–<18 months stratified by breastmilk intake, OzFITS 2021.

Food Group	Consumers *n* (%)	Energy, kJ/day [1]	Percentage of total energy intake [1]
**Breastfed toddlers (*n* = 119)**			
Fruit	116 (97)	313 (138–524)	8 (4–13)
Vegetables	118 (99)	151 (30–378)	4 (1–9)
Cereals and grains	115 (97)	654 (327–1057)	18 (10–25)
Meats and alternatives	111 (93)	286 (71–689)	8 (3–16)
Dairy			
Without toddler milks	107 (90)	417 (110–743)	11 (3–19)
With toddler milks [2]	107 (90)	432 (110–792)	11 (3–21)
Fats and oils	69 (58)	37 (0–185)	1 (0–4)
Discretionary foods	101 (85)	290 (85–737)	8 (2–18)
**Non-breastfed toddlers (*n* = 114)**			
Fruit	108 (95)	405 (249–622)	10 (6–14)
Vegetables	111 (97)	188 (58–464)	5 (1–11)
Cereals and grains	110 (96)	880 (514–1237)	22 (12–30)
Meats and alternatives	102 (89)	335 (59–750)	9 (2–17)
Dairy			
Without toddler milks	113 (99)	1065 (600–1517)	24 (15–38)
With toddler milks [2]	114 (100)	1499 (1013–1896)	37 (24–49)
Fats and oils	67 (59)	23 (0–139)	1 (0–4)
Discretionary foods	100 (88)	331 (82–841)	9 (2–20)

[1] Values are median (IQR). [2] Dairy, including formula/toddler milk. A serve of formula/toddler milk is defined as 250 mL prepared volume.

Table S3. Daily serves of five food group and discretionary foods consumed by toddlers 18–24 months stratified by breastmilk intake, OzFITS 2021.

Food Group	Consumers *n* (%)	AGHE Serve size [1]	Recommended serves per day	Number of serves per day [2]
**Breastfed toddlers (*n* = 90)**				
Fruit	86 (96)	150 g	½	0.89 (0.48–1.62)
Vegetables	86 (96)	75 g	2–3	1.08 (0.49–2.40)
Cereals and grains	90 (100)	40 g bread [1]	4	2.01 (1.0–3.13)
Meats and alternatives	82 (91)	65 g red meat [1]	1	0.55 (0.25–1.29)
Dairy				
Without toddler milks	83 (92)	250 ml milk [1]	1–1 ½	0.73 (0.42–1.42)
With toddler milks [3]	83 (92)	250 ml milk [1]	1–1 ½	0.76 (0.43–1.47)
Fats and oils	54 (60)	7–10 g	1	0.24 (0–0.57)
Discretionary foods	79 (88)	418 kJ	0	1.37 (0.41–2.52)
**Non-breastfed toddlers (*n* = 152)**				
Fruit	147 (97)	150 g	½	1.25 (0.58–1.85)
Vegetables	143 (94)	75 g	2-3	1.22 (0.56–2.61)
Cereals and grains	151 (99)	40 g bread [1]	4	2.54 (1.71–3.39)
Meats and alternatives	137 (90)	65 g red meat [1]	1	0.62 (0.24–1.19)
Dairy				
Without toddler milks	147 (97)	250 mL milk [1]	1–1 ½	1.24 (0.85–1.93)
With toddler milks [3]	151 (99)	250 mL milk [1]	1–1 ½	1.66 (0.93–2.40)
Fats and oils	108 (71)	7–10 g	1	0.31 (0–0.89)
Discretionary foods	142 (93)	418 kJ	0	1.51 (0.60–2.68)

[1] AGHE; Australian Guide to Healthy Eating; Equivalent of 40 g bread, 65 g red meat, or 250 ml milk. For example, 40g of cheese equals one serve of dairy [1]. [2] Values are median (IQR). The lower bound is used where a range exists for a recommended serves per day. [3] Dairy, including formula/toddler milk. A serve of formula/toddler milk is defined as 250 mL prepared volume.

Table S4. Energy and percentage total energy from five food group and discretionary foods for toddlers 18–24 months stratified by breastmilk intake, OzFITS 2021.

Food Group	Consumers *n* (%)	Energy, kJ/day [1]	Percentage of total energy intake [1]
**Breastfed toddlers (*n* = 90)**			
Fruit	86 (96)	294 (158–557)	6 (4–12)
Vegetables	86 (96)	146 (24–324)	3 (1–1)
Cereals and grains	90 (100)	836 (417–1394)	19 (10–31)
Meats and alternatives	82 (91)	500 (150–843)	11 (3–19)
Dairy			
Without toddler milks	83 (92)	520 (203–1004)	14 (6–23)
With toddler milks [2]	83 (92)	520 (205–1021)	14 (6–24)
Fats and oils	54 (60)	58 (0–148)	1 (0–3)
Discretionary foods	79 (88)	575 (172–1056)	12 (4–25)
**Non-breastfed toddlers (*n* = 152)**			
Fruit	147 (97)	415 (209–645)	9 (5–14)
Vegetables	143 (94)	185 (19–476)	4 (1–12)
Cereals and grains	151 (99)	1003 (620–1434)	23 (14–32)
Meats and alternatives	137 (90)	417 (122–858)	11 (3–20)
Dairy			
Without toddler milks	147 (97)	823 (470–1328)	20 (11–30)
With toddler milks [2]	151 (99)	1129 (630–1562)	26 (16–37)
Fats and oils	108 (71)	82 (0–218)	2 (0–5)
Discretionary foods	142 (93)	633 (249–1121)	16 (6–26)

[1] Values are median (IQR). [2] Dairy, including formula/toddler milk. A serve of formula/toddler milk is defined as 250 mL prepared volume.
